# A unifying nonlinear probabilistic epidemic model in space and time

**DOI:** 10.1038/s41598-021-93388-1

**Published:** 2021-07-05

**Authors:** Roberto Beneduci, Eleonora Bilotta, Pietro Pantano

**Affiliations:** 1 Department of Physics, University of Calabria, Rende, CS 87036 Italy; 2 Istituto Nazionale di Fisica Nucleare, gruppo collegato Cosenza, Rende, CS 87036 Italy

**Keywords:** Applied mathematics, Systems biology, Diseases

## Abstract

Covid-19 epidemic dramatically relaunched the importance of mathematical modelling in supporting governments decisions to slow down the disease propagation. On the other hand, it remains a challenging task for mathematical modelling. The interplay between different models could be a key element in the modelling strategies. Here we propose a continuous space-time non-linear probabilistic model from which we can derive many of the existing models both deterministic and stochastic as for example SI, SIR, SIR stochastic, continuous-time stochastic models, discrete stochastic models, Fisher–Kolmogorov model. A partial analogy with the statistical interpretation of quantum mechanics provides an interpretation of the model. Epidemic forecasting is one of its possible applications; in principle, the model can be used in order to locate those regions of space where the infection probability is going to increase. The connection between non-linear probabilistic and non-linear deterministic models is analyzed. In particular, it is shown that the Fisher–Kolmogorov equation is connected to linear probabilistic models. On the other hand, a generalized version of the Fisher–Kolmogorov equation is derived from the non-linear probabilistic model and is shown to be characterized by a non-homogeneous time-dependent diffusion coefficient (anomalous diffusion) which encodes information about the non-linearity of the probabilistic model.

## Introduction

In December 2019, the novel SARS-CoV-2 (that shaped the Covid-19 disease) was detected in a patient working in the fish reservoir of Wuhan (Hubei, China). The virus has produced 118268575 infected and 2624677 deaths (World Health Center, 12 March 2021), thus creating a pandemic spread worldwide. While national governments took steps to protect people with extreme safety measures, it was soon understood that the SARS-CoV-2 virus is characterized by a peculiar behaviour compared with previous coronaviruses^1, 2, 4^ opening several scientific questions about how to model the SARS-CoV-2 virus. Some of its peculiarities are connected with: long incubation period, role of the asymptomatic individuals^3^, intensity of transmission^5, 6^ possible role of kids in the contagion. That dramatically called attention to the relevance of mathematical modeling for the description and the forecasting of the epidemic and, most importantly, relaunched its relevance in supporting governments decisions about the kind of measures to be activated and the right choice of their timing in order to slow down the disease propagation.

Several factors should play a role in modelling epidemics in general. Some of those factors becomes particularly relevant in the case of the Covid-19 epidemic as for example spatial heterogeneity^7–9^, heterogeneity of population^10^ (age, health conditions, etc.) and role of susceptible^3^. In particular, spatial heterogeneity assumes a key role in coping with COVID-19 epidemic. Indeed, mathematical models are being used by governments to make decisions about the quarantine measures to be adopted in order to slow down the epidemic evolution. In its turn, quarantine is inevitably connected to the economic dynamics of nations and regions and the world is witnessing the enormous damages that the lockdown (which has been necessary in order to stop the epidemic evolution) has caused. In this framework, spatial models assume a key role since they can be used to differentiate the lockdown measures in the different regions (and/or cities) of a given country. That would potentially reduce the economic damages.

Scientific studies on Epidemics started from the ground-breaking works of Bernoulli^11^ (1760), En’ko (1889), (reported in Dietz^12^), Ross^13^ (1911), Lotka (1923), Kostitzin (1934), Volterra (1938) (reported in Scudo^14^), Kermack and McKendrick^15^ (1927) and then a lot of mathematical models ranging from stochastic to deterministic models (see Bauer^16^; Bailey^17^; Diekmann^18^; Isham^19^; Keeling and Rohani^20^; Riley^21^ for systematic expositions).

Despite the increasing number of important papers^5, 22–32^ (just to cite some of them) on the mathematical modelling of the novel COVID-19 pandemic, a general theoretical framework to model and forecast the epidemic evolution is still lacking. It has been pointed out that the choice of a model (for example, deterministic or stochastic) depends, among others, on the epidemic under investigation, on the scale one decides to focus on as well as on the evolution step of the epidemic. Recently^31^ it has been suggested that a good strategy in the modelling of Covid-19 epidemics would be to use different types of parsimonious models at the different steps of the epidemic. That would require an understanding of the relationships between the different models. Several efforts have been devoted to this problem^33–35^. Moreover, it has been observed that understanding the limit of a model is a very important aspect of mathematical modelling and that a model too rich in details is not helpful if data are uncertain and/or the model assumptions are not adequate to the phenomenology it aims to describe^36, 37^. All of that underlines the relevance of a unified approach to epidemic modelling. Here we propose a novel approach which is inspired by the statistical interpretation of quantum mechanics^39^ and that goes in this direction. In particular we propose a continuous non-linear space-time model of the epidemic embodied into a probability transition kernel that includes as particular cases several of the most important epidemic models as for example SIR, SI, time-continuous stochastic models, time-discrete stochastic models, SIR stochastic model and diffusion models. In particular a generalized version of the Fisher–Kolmogorov model displaying anomalous diffusion is derived.

The approach we propose in the present paper should be interesting in itself from the theoretical viewpoint since all those models are shown to be particular cases of a “mother model” from which they are derived under precise assumptions but it could also be relevant to epidemic forecasting (see next section) and in order to deal with the problems we outlined above, i.e., using different models simultaneously, choosing the appropriate model to be used at the different steps of the epidemic (the choice being linked to the approximation needed to derive the model from the “mother model”), pinpointing the limit of the various models. Those are problems deserving a future work; here we limit ourselves to present the model and its potentialities and to show how several other models can be derived from it. For example, we provide a connection between diffusion and probabilistic models and show that more general versions of the Fisher–Kolmogorov equation are connected to probabilistic non-linear models. Moreover, the two-dimensional Fisher–Kolmogorov equation is derived from a linear version of the probabilistic mother model (which is obtained by assuming the probability density of susceptible to be constant). This last result confirms and extends the results in Mollison^34^ where it is shown that the non-linearity of the Fisher–Kolmogorov model is not sufficient to fully encode the non-linearity of the stochastic model it aims to emulate^33^. Anyway it seems to be relevant that more general version of the Fisher–Kolmogorov equation are connected to non-linear probabilistic models. Later (see the comment after Eq. (15)) the time dependence of the diffusion coefficient is shown to play a role in encoding the non-linearity. That is of general physical interest. We recall that the lack of an exact and clear derivation of the Fisher–Kolmogorov equation has been pointed out^33^. Finally, it has been observed^34^ that in order to justify the use of deterministic models, the latter should be interpreted as approximations of stochastic models and not the other way around. Our results should be relevant to this problems as well. Although the present work suggests a general theoretical framework for epidemic modelling, it has been motivated by the Covid-19 emergency that we use as a reference point for our argumentation. Potential applications of the model concern the localization of those regions of space where the infection probability is going to increase (see next section); that will be the topic of a future work.

## Results

### The probabilistic model

**Figure 1:**
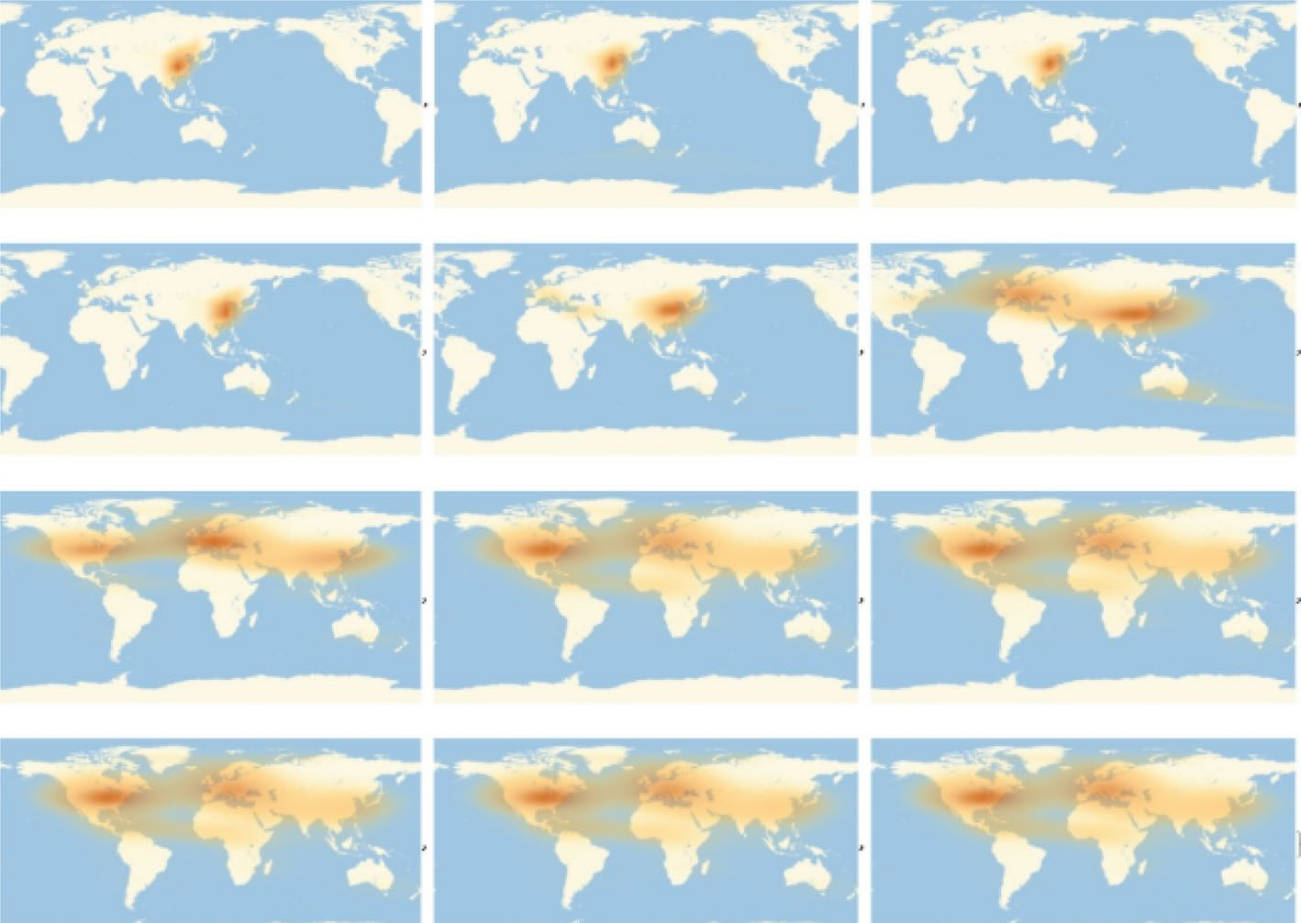
Qualitative illustration of the spatio-temporal evolution of COVID-19. The software Wolfram Mathematica (version 11.3) has been used to estimate $\psi ^I$ from the data about the space distribution of the infected. The software uses the kernel density estimation method^38^ to estimate $\psi ^I$. Then, the same software has been used in order to generate a sampling, i.e., a set of points on the surface $\Sigma$ distributed according to $\psi ^I$. That has been done at several instant of time. The image illustrates qualitatively the expansion of the infected population. The infected cloud on the ocean is an artifact.

In order to illustrate the rationale of the model we can look at Fig. 1 which shows the spread of the COVID-19 on a global scale from the 22th of February to the 20th of May 2020. A logarithm density plot of the number of infected people using a geo-referenced map is shown in Fig. 1 which highlights the east-west direction of the virus propagation from China to Europe and then to America, with an increasing propagation of the outbreak. Let $\Sigma$ be the surface of the earth we focus on. A point in the surface is denoted by $\mathbf {x}=(x,y)\in \Sigma$. We assume there are positive definite functions $\psi ^I_t(\mathbf {x}),\psi ^R_t(\mathbf {x}),\psi ^S_t(\mathbf {x}),\in L^1(\Sigma ,d^2\mathbf {x})$ representing sub-probability densities and describing the state of the epidemic at time *t*. In particular, $\psi _t^I(\mathbf {x})$ is the sub-probability density for the infected, $\psi _t^R(\mathbf {x})$ the sub-probability density for the recovered (infected which turn into immune) and $\psi ^S_t(\mathbf {x})$ the sub-probability density for the susceptible. The probability density functions are assumed to be derivable with respect to the time variable. Moreover, we assume $\psi ^S_0(\mathbf {x})+\psi ^I_0(\mathbf {x})=\psi ^S_t(\mathbf {x})+\psi ^I_t(\mathbf {x})+\psi ^R_t(\mathbf {x})$ for every *t* and $\int _\Sigma (\psi ^S_0(\mathbf {x})+\psi ^I_0(\mathbf {x}))\,d\mathbf {x}=1$. As a consequence we are not considering births and deaths. It is worth noticing that the existence of $\psi _t^I$, $\psi _t^R$ and $\psi ^S_t$ is a theoretical assumption; the actual infected distribution is just an instantiation of the random process associated to the infected distribution. Anyway, an approximation of $\psi _t^I$, $\psi _t^R$ and $\psi ^S_t$ can be obtained through density estimation methods as for example the kernel density estimation^38^ (KDE) which provides a differentiable probability density function.

We suggest a partial analogy with the interpretation of quantum mechanics^39^ where the state of a particle is described by a probability cloud which is given by the square of the modulo of the wave function $\Psi$. In particular, $|\Psi (t,x)|^2\in L^2(\mathbb {R})$ is a probability density function and $\int _\Delta |\Psi (t,x)|^2\,dx$ is interpreted as the probability that the particle is found to be in $\Delta$ at time *t*. No further specification is required. Analogously, the state of the epidemic is defined by three probability clouds $(\psi ^I,\psi ^S,\psi ^R)$ and $\int _{\Delta }\psi ^I_t(\mathbf {x})\,d^2\mathbf {x}$ is interpreted as the probability that an infected is in the region $\Delta \subset \Sigma$ at time *t*. No further specification will be required in the present approach. The statistical interpretation of quantum mechanics^39^ introduces the concept of ensemble of identical copies of the particle in order to explain the meaning of the function $\Psi$. To be more precise, $\Psi$ describes an infinite ensemble of copies of the particle; each copy being prepared by the same experimental arrangement. The function $|\Psi |^2$ then gives the statistical distribution of the position of the particle among the copies of the conceptual ensemble. We can adapt such an interpretation in the present context by interpreting $\psi ^I$ as follows: suppose there is a mechanism which encodes the nature of the epidemic as well as the contingent circumstances it is determined from; suppose such a mechanism can be used to produce an individual; he/she will be an infected with probability $\int _\Sigma \psi _t^I(\mathbf {x})$, a susceptible with probability $\int _\Sigma \psi _t^S(\mathbf {x})$ and a recovered with probability $\int _\Sigma \psi _t^R(\mathbf {x})$; think of an infinite ensemble of copies of the same production mechanism each one producing an individual. Then $\psi ^I$, $\psi ^S$, $\psi ^R$ give respectively the space distribution of the infected, susceptible and recovered among the copies of the ensemble and $\int _\Delta \psi _t^I(\mathbf {x}\,d^2\mathbf {x}$ is interpreted as the probability that a copy of the ensemble is made of an infected located in the set $\Delta$. That provides a possible interpretation of the state of the epidemic as we have defined it and is helpful, we think, in order to explains the role that $\psi ^I$ plays in the present paper.

In quantum mechanics, the dynamics of the system is determined by a deterministic evolution equations for $\Psi$ (the Schroedinger equation). Pushing further the analogy with quantum mechanics, the dynamics of the epidemic is given by the evolution equations for $\psi ^I$ and $\psi ^S$ [see Eq. (6) and (8)]. Note that a deterministic evolution for the probability density does not mean that the epidemic evolves deterministically. It is a probabilistic process.

One could ask what is the connection between the state of the epidemic as we have defined it and the actual observation of the epidemic. Suppose we are able to generate an individual as many times as we want as we discussed previously. Suppose we use such a generation procedure to provide a family of ensembles $\mathcal {E}_1,\dots \mathcal {E}_k$ each one made of *N* copies of the individual according to the state ($\psi ^I_t$, $\psi ^S_t$, $\psi ^R_t$). The ensembles $\mathcal {E}_i$ can be considered as instantiations of the epidemic with *N* individuals. The distribution of the infected, $\sigma _j^I$ (number of infected over surface), corresponding to the instantiation $\mathcal {E}_j$ is in general different from the distribution of the infected $\sigma ^I_i$ corresponding to $\mathcal {E}_i$. Despite the number of infected (susceptible) in a given region will be different in any instantiation, the expected number of infected (susceptible) in a given region $\Delta$ is meaningful from the statistical viewpoint and is given by $N\int _{\Delta }\psi ^I_t(\mathbf {x})\,d^2\mathbf {x}$ ($N\int _{\Delta }\psi ^S_t(\mathbf {x})\,d^2\mathbf {x}$). By the law of large numbers, the probability that the number of infected in $\mathcal {E}_i$ differs from $N\int _{\Delta }\psi ^I_t(\mathbf {x})\,d^2\mathbf {x}$ can be made arbitrarily small by choosing *N* sufficiently large.

We remark that in the present paper the focus is on the probability density functions $\psi ^I_t$, $\psi ^S_t$, $\psi ^R_t$ which, by analogy with quantum mechanics, define the state of the epidemic. The approach is then phenomenological as opposed to mechanistic; we assume the existence of probability density functions describing the probabilistic distribution of infected, susceptible, and recovered (the state of the epidemic) and limit ourselves to describe their time evolution for which we postulate a deterministic law (see below). The main aim of the present paper is to show that this approach defines a non-linear probabilistic model from which many of the existing models can be derived providing at the same time a unifying framework.

Moreover, the model could be used to describe the evolution of the infected spatial probability density in order to locate those regions of space where the probability of infection is going to increase or to decrease. That could be very helpful to governments as we remarked in the introduction and will be the topic of a future paper.

Now, we proceed to define the evolution equation for the state of the epidemic. We provide a non-linear evolution for the sub-probability distributions.

In the following, $\alpha (t)$ denotes the probability rate that infected turn to immune; it is assumed to be continuous and such that, $\int _0^t\alpha (\tau )\,d\tau =p(t)\le 1$, for all $t\in \mathbb {R}^+$, where *p*(*t*) is the probability that an infected turns into immune during the time interval [0, *t*]. The probability rate $\alpha$ can be used to connect $\psi ^R$ and $\psi ^I$: $\psi ^R_t(\mathbf {x}_2)=\int _0^t\alpha (\tau )\psi ^I_\tau (\mathbf {x_2})d\tau$. The space dependence of $\alpha$ can also be included if necessary. Moreover we assume there are no births and deaths and no displacements of the individuals (see the comments after Eq. (3) and before Eq. (5) for further details). The epidemic propagation is encoded into a kernel $W(t,\mathbf {x_2},\mathbf {x_1})$ which describes the transition probability rate of the infection from $\mathbf {x_1}$ to $\mathbf {x_2}$ at time *t*. In particular,

$$\begin{aligned}{}[d^2\mathbf {x}_2\psi ^S_\tau (\mathbf {x}_2)W(\tau ,\mathbf {x}_2,\mathbf {x}_1)d\tau ] \end{aligned}$$

is interpreted as the conditional probability that a susceptible in the neighbourhood centered in $\mathbf {x}_2$ with radius $d^2\mathbf {x}_2$ gets infected during the time interval $[\tau ,\tau +d\tau ]$ if an infected is present in $\mathbf {x}_1$ at time $\tau$. Then,

1 $$\begin{aligned}{}[d^2\mathbf {x}_2\psi ^S_\tau (\mathbf {x}_2)W(\tau ,\mathbf {x}_2,\mathbf {x}_1)d\tau ](\psi ^I_\tau (\mathbf {x}_1)\,d^2\mathbf {x}_1) \end{aligned}$$

is interpreted as the probability that a susceptible in the neighbourhood centered in $\mathbf {x}_2$ with radius $d^2\mathbf {x}_2$ gets infected by the infected contained in the neighbourhood centered in $\mathbf {x}_1$ with radius $d^2\mathbf {x}_1$ during the time interval $[\tau ,\tau +d\tau ]$. Hence,

2 $$\begin{aligned} d^2{\mathbf {x}_2}\int _{\Sigma }\int _0^{t}\,\psi ^S_\tau (\mathbf {x}_2)W(\tau ,\mathbf {x}_2,\mathbf {x}_1)\psi ^I_\tau (\mathbf {x}_1)\,d\tau \,d^2\mathbf {x}_1 \end{aligned}$$

gives the probability of new infected in the neighbourhood centered in $\mathbf {x}_2$ with radius $d^2\mathbf {x}_2$ during the time interval [0, *t*]. Since in the same time interval there is a probability that the infected turn into recovered, we have

3 $$\begin{aligned} d^2{\mathbf {x}_2}\int _{\Sigma }\int _0^{t}\,\psi ^S_\tau (\mathbf {x}_2)W(\tau ,\mathbf {x}_2,\mathbf {x}_1)\psi ^I_\tau (\mathbf {x}_1)\,d\tau \,d^2\mathbf {x}_1=d^2{\mathbf {x}_2}[\psi ^I_t(\mathbf {x}_2)-\psi ^I_0(\mathbf {x}_2)+\psi ^R_t(\mathbf {x}_2)]. \end{aligned}$$

where $\psi ^R_t(\mathbf {x}_2)=\int _0^t\alpha (\tau )\psi ^I_\tau (\mathbf {x_2})d\tau$. The previous probabilities can be interpreted by resorting to the idea of conceptual ensemble we introduced above. In particular, one can think of the ensemble of infinite copies of a single individual with each copy generated according to the state of the epidemic by the procedure described above; each individual being fixed in his/her position with the susceptible that can become infected and the infected that can become recovered according to the probabilities introduced above. In the case of an instantiation of the epidemic with *N* individuals fixed in their positions and supposing *N* sufficiently large, the evolution of the population densities ($\sigma ^I$, $\sigma ^S$, $\sigma ^R$) can be approximated by using the previous probabilities (see also Fig. 3 on this last point).

**Figure 2:**
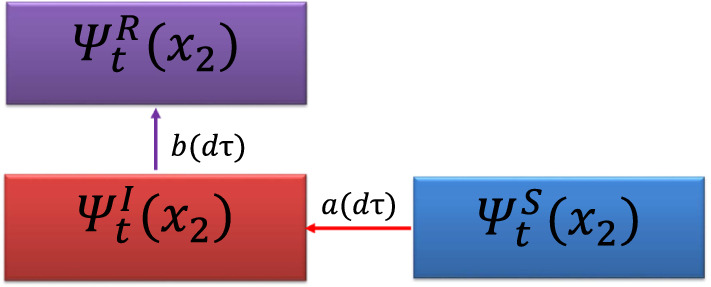
Evolution of the sub-probability densities during an infinitesimal time interval $d\tau$. Here, $a\,d\tau =\int _{\Sigma }\psi ^S_\tau (\mathbf {x}_2)W(\tau ,\mathbf {x}_2,\mathbf {x}_1)\psi ^I_\tau (\mathbf {x}_1)d^2\mathbf {x}_1\,d\tau$ and $b\,d\tau =\alpha (\tau )\psi ^I_\tau (\mathbf {x_2})d\tau$. The arrows represent the direction of the probability fluxes.

Note that, at variance with the second member of Eq. (3), the first member does not depend on $\alpha$. The role of $\alpha$ is to give the probability that infected turn into recovered while the first member gives the probability of new infected which depends on $\alpha$ only indirectly: a higher $\alpha$ means a lower $\psi _\tau ^I$. The flux diagram in Fig. 2 illustrates the variation of the sub-probability distributions during an infinitesimal time interval $d\tau$.

By (3) we obtain,

4 $$\begin{aligned} \psi ^I_t(\mathbf {x}_2)&=\psi ^I_0(\mathbf {x}_2)-\psi ^R_t(\mathbf {x}_2)+\int _{\Sigma }\int _0^{t}\,\psi ^S_\tau (\mathbf {x}_2)W(\tau ,\mathbf {x}_2,\mathbf {x}_1)\psi ^I_\tau (\mathbf {x}_1)\,d\tau \,d^2\mathbf {x}_1\nonumber \\&=\psi ^I_0(\mathbf {x}_2)-\int _0^t\alpha (\tau )\psi ^I_\tau (\mathbf {x}_2)\,d\tau +\int _{\Sigma }\int _0^{t}\,\psi ^S_\tau (\mathbf {x}_2)W(\tau ,\mathbf {x}_2,\mathbf {x}_1)\psi ^I_\tau (\mathbf {x}_1)\,d\tau \,d^2\mathbf {x}_1 \end{aligned}$$

with,

$$\begin{aligned} \psi ^S_0(\mathbf {x})+\psi ^I_0(\mathbf {x})&=\psi ^S_t(\mathbf {x})+\psi ^I_t(\mathbf {x})+\psi ^R_t(\mathbf {x})\\ \int _\Sigma \big (\psi ^S_0(\mathbf {x})+\psi ^I_0(\mathbf {x})&=\psi ^S_t(\mathbf {x})+\psi ^I_t(\mathbf {x})+\psi ^R_t(\mathbf {x})\big )\,d^2\mathbf {x}=1. \end{aligned}$$

One can think of

$$\begin{aligned} \mu _{\Delta _2}(\tau ,\mathbf {x}_1)\,d\tau :=\int _{\Delta _2}[W(\tau ,\mathbf {x}_2,\mathbf {x}_1)d\tau ]\psi ^S_\tau (\mathbf {x}_2)d^2\mathbf {x}_2 \end{aligned}$$

as the transition probability that a susceptible in $\Delta _2$ gets infected during the time interval $(\tau ,\tau +d\tau )$ if in $\mathbf {x}_1$ there is an infected. Note that $\mu _{(\cdot )}(\tau ,\mathbf {x}_1)$ is absolutely continuous with respect to $\int _{(\cdot )}\psi ^S_\tau (\mathbf {x}_2)\,d\mathbf {x}_2$ and $W(\tau ,\mathbf {x}_2,\mathbf {x}_1)$ is the the Radon-Nikodym derivative of $\mu _{(\cdot )}(\tau ,\mathbf {x}_1)$ with respect to $\int _{(\cdot )}\psi ^S_\tau (\mathbf {x}_2)\,d\mathbf {x}_2$. That seems quite natural since $\int _{\Delta _2}\psi ^S_\tau (\mathbf {x}_2)\,d\mathbf {x}_2=0$ implies that no susceptible can be infected inside $\Delta _2$. Moreover, it provides an interpretation in terms of transition probability and explains the role that the susceptible probability density $\psi ^S$ plays in the definition of *W*.

It is worth remarking that the kernel is intended to provide a phenomenological description of the evolution. We are assuming there are no displacement of the individuals. We just assume the existence of the transition probability (1).

Supposing $\epsilon$ sufficiently small, we can use (4) to derive the evolution from the state at time *t* to the state at time $t+\epsilon$,

5 $$\begin{aligned} \psi ^I_{t+\epsilon }(\mathbf {x}_2)&=\int _{\Sigma }\int _0^{t+\epsilon }\,K(\tau ,\mathbf {x}_2,\mathbf {x}_1)\psi ^I_\tau (\mathbf {x}_1)\,d\tau \,d^2\mathbf {x}_1 \nonumber \\&=\int _{\Sigma }\int _0^{t}\,K(\tau ,\mathbf {x}_2,\mathbf {x}_1)\psi ^I_\tau (\mathbf {x}_1)\,d\tau \,d^2\mathbf {x}_1+\int _{\Sigma }\int _t^{t+\epsilon }\,K(\tau ,\mathbf {x}_2,\mathbf {x}_1)\psi ^I_\tau (\mathbf {x}_1)\,d\tau \,d^2\mathbf {x}_1\nonumber \\&=\psi ^I_t(\mathbf {x_2})-\alpha (t)\psi ^I_t(\mathbf {x}_2)\,\epsilon +\epsilon \psi ^S_t(\mathbf {x}_2)\int _{\Sigma }\,W(t,\mathbf {x}_2,\mathbf {x}_1)\psi ^I_t(\mathbf {x}_1)\,d^2\mathbf {x}_1 \end{aligned}$$

Then, we can derive an integro-differential equation for the infected sub-probability density $\psi ^I_t$. Indeed $\psi ^I_{t+\epsilon }(\mathbf {x}_2)=\psi ^I_t(\mathbf {x_2})+\epsilon \frac{\partial \psi ^I_t(\mathbf {x}_2)}{\partial t}+o(\epsilon )$ so that

6 $$\begin{aligned} \frac{\partial \psi ^I_t(\mathbf {x}_2)}{\partial t}=-\alpha (t)\psi ^I_t(\mathbf {x}_2)+\psi ^S_t(\mathbf {x}_2)\int _{\Sigma }\,W(t,\mathbf {x}_2,\mathbf {x}_1)\psi ^I_t(\mathbf {x}_1)\,d^2\mathbf {x}_1 \end{aligned}$$

In order to derive the differential equation for the susceptible sub-probability density we write the susceptible sub-probability density at time $t+\epsilon$ as a function of the susceptible sub-probability density at time *t*,

7 $$\begin{aligned} \psi ^S_{t+\epsilon }(\mathbf {x}_2)=\psi ^S_{t}(\mathbf {x}_2)-\int _\Sigma \int _t^{t+\epsilon } \psi ^S_{\tau }(\mathbf {x}_2)W(\tau ,\mathbf {x}_2,\mathbf {x}_1)\,\psi ^I_\tau (\mathbf {x}_1)\,d^2\mathbf {x}_1d\tau \end{aligned}$$

where the first term in the second member is the sub-probability density at time *t* and the second term is the probability density of the new infected produced in $\mathbf {x}_2$ in the time interval $[t,t+\epsilon ]$. Then, by using the Taylor expansion for $\psi ^S_{t+\epsilon }(\mathbf {x}_2)$, we obtain

8 $$\begin{aligned} \frac{\partial \psi ^S_{t}(\mathbf {x}_2)}{\partial t}=-\psi ^S_{t}(\mathbf {x}_2)\int _{\Sigma }W(t,\mathbf {x}_2,\mathbf {x}_1)\psi ^I_t(\mathbf {x}_1)d^2\mathbf {x}_1. \end{aligned}$$

**Figure 3:**
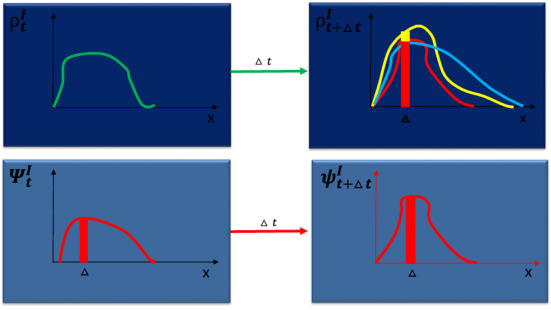
The upper part of the figure illustrates a hypothetical evolution of the normalized density of infected $\rho ^I=\sigma ^I/N$ (density of infected over the total number of individuals) from time *t* to time $t+\Delta t$ and shows that during the time interval $\Delta t$ it can evolve in different possible ways because of the probabilistic nature of the system. That is not the case of the sub-probability density $\psi ^I$ whose evolution is unique (lower part of the figure). Note also that $N\int _\Delta \psi _t^I(x)\,dx$ can be very different from the number of infected contained in $\Delta$ at time *t* (compare, for example, the yellow curve in the upper part of the figure with the red curve in the lower part). Only in the case *N* is very large (deterministic limit of the model) the evolution of the normalized density $\rho ^I$ follows, with a high probability, the evolution of the probability density $\psi ^I$ and the probability that $N\int _\Delta \psi _t^I(x)\,dx$ coincides with the number of infected in the region $\Delta$ at time *t* is high. Going back to the upper part of the figure, it is worth remarking that the deterministic models choose one of the possible evolutions for the normalized density of infected, $\rho ^I$. For example, in the Kendall model^40^ a system of differential equations for $\rho _t^I$ is postulated; it is equivalent (up to some restrictions on the structure of the kernel: the kernel is not time dependent and depends on the difference $\mathbf {x}-\mathbf {y}$) to the system of differential equations for the probability density $\psi ^I_t$ we derived in the present work; such a postulate is sound only if *N* is sufficiently large.

It is interesting to note that an equation similar to Eq. (6) and describing the evolution of the total size of an infective population $I(t,\xi )$ in a deterministic framework can be found in Diekmann^18^ (see exercise 8.21, page 219). It is derived in a different framework where $I(t,x)=\int _{-\infty }^te^{-\alpha (x)(t-\tau )}i(\tau ,x)\,d\tau$ and *i* is the expected number of new infected per unit of time. The kernel *A*(*t*, *x*, *y*) defines the evolution of *i*. In particular, $i(t,x)=S(t,x)\int _0^\infty \int _\Sigma A(\tau ,x,y)i(t-\tau ,y )\,dy\,d\tau$ where *S* denotes the density of susceptible and a particular form of the kernel is assumed, $A(t,x,y)=\beta (x,y)e^{-\alpha \tau }$. Note that in the present paper *i* assumes the form $\int _\Sigma \psi ^S_\tau (\mathbf {x})W(\tau ,\mathbf {x},\mathbf {y})\psi ^I_\tau (\mathbf {y})\,d^2\mathbf {y}$. An equivalent, less general, system of differential equations for the evolution of the normalized densities $\rho ^I_t=\sigma ^I/N$ and $\rho ^S_t=\sigma ^S/N$ of infected and the susceptible (density over the total number of individuals) in a deterministic model can be found in Kendall^40^. For *N* very large we recover a generalized version of the Kendall model since $\rho ^I$, $\rho ^I$, $\rho ^R$ provide an approximation of the sub-probability densities $\psi ^I$, $\psi ^S$ and $\psi ^R$. Therefore, Kendall’s model can be considered as a particular deterministic limit of the probabilistic model we suggest (see Fig. 3 for more details). An equation similar to Eq. (8) can be found in the deterministic model proposed by Thieme^41^ which is based on a different approach and where the kernel $\hat{k}$ differs from *W* and assumes a different meaning. In particular, $i(t,x)=\int _\Sigma \int _0^t\int _0^\infty I(t-\tau ,c,y)\,\hat{k}(x;dy\,dc\,d\tau )$ where *i* is defined as the infective influence, *I* is the density of the infected and *c* denotes the age class. Then, the differential equation for the susceptible takes the form $\frac{\partial \psi ^S_t(\mathbf {x})}{\partial t}=\eta (\psi _t^I(\mathbf {x}))\,i(t,\mathbf {x})$ which compared with Eq. (8) shows the differences between $\hat{k}$ and *W*. We remark that, at variance with the space models just cited, in the present paper, Eqs. (6) and (8) refer to a probabilistic model and take on a particular meaning which is connected to the evolution of the sub-probability density functions describing the epidemic. They are assumed to grasp the law governing the evolution of the epidemic and then to provide the exact description of its evolution. We are assuming the existence of such a law which is encoded into the kernel *W*. Moreover, the integro-differential Eqs. (6) and (8) are derived in a probabilistic framework based on conditional probabilities. The probabilistic nature of the model and the differences with deterministic models is depicted in Fig. 3 below.

Extending further the analogy with quantum mechanics we can think of *W* as the analogous of the Hamiltonian operator in quantum mechanics which describes the interaction between the different particles of the system. Here *W* takes into account the interaction between infected and susceptible.

Note that Eqs. (6) and (8) define a non linear system of differential equations. The probabilistic framework we introduced is very general and is shown to contain several well known models that can be derived from it. We start with a generalized version of the Fisher–Kolmogorov model (Eq. (14)). Next we discretize the space coordinate and obtain a model which is continuous in time and discrete in space (Eqs. (16) and (18)). By neglecting the space effects, we derive the SIR and SI models (Eqs. (29) and (31)). By discretization of the time coordinate we obtain a general time-discrete stochastic model (Eq. (20)) from which the SIR stochastic model can be derived (Eq. 22).

### Derivation of a generalized version of the Fisher–Kolmogorov model

The Fisher–Kolmogorov equation

9 $$\begin{aligned} \frac{\partial \rho }{\partial t}-D\frac{\partial ^2\rho }{\partial x^2}=\eta (1-\rho )\rho \end{aligned}$$

where $\rho _t(x)$ denotes the density (or frequency) of a population is a particular kind of drift-diffusion equation that has been used in order to describe the frequency of a mutant gene in a population^42^ (Fisher 1937). Later, a more general version has been studied by Kolmogorov^43^ et al. (1937). We derive a generalization of the Fisher–Kolmogorov equation for the infected probability density $\psi ^I$ that, assuming the stochastic fluctuations can be neglected, can be used to derive the evolution of the infected density of a population of *N* individuals. Then, under some stronger hypothesis, we derive the 2-dimensional version of Eq. (9).

Let us suppose that $W(t,\mathbf {x}_2,\mathbf {x}_1)$ depends only on the distance between $\mathbf {x}_2$ and $\mathbf {x}_1$ and $\Sigma =\mathbb {R}^2$. Then Eq. (5) becomes

10 $$\begin{aligned} \psi ^I_{t+\epsilon }(\mathbf {x}_2)&=\psi ^I_t(\mathbf {x}_2)-\alpha (t)\psi ^I_t(\mathbf {x}_2)\,\epsilon +\epsilon \psi ^S_t(\mathbf {x}_2)\int _{\Sigma }\,W(|\mathbf {x}_2-\mathbf {x}_1|)\psi ^I_t(\mathbf {x}_1)\,d^2\mathbf {x}_1\nonumber \\&=\psi ^I_t(\mathbf {x}_2)-\alpha (t)\psi ^I_t(\mathbf {x}_2)\,\epsilon +\epsilon \psi ^S_t(\mathbf {x}_2)\int _{\Sigma }\,W(|\mathbf {z}|)\psi ^I_t(\mathbf {x}_2-\mathbf {z})\,d^2\mathbf {z} \end{aligned}$$

where $\mathbf {z}=\mathbf {x}_2-\mathbf {x}_1$. Furthermore suppose that in the time interval $\epsilon$, $\epsilon W$ is different from zero only for very small distances, $|\mathbf {z}|$, and consider the second order Taylor expansion of $\psi ^I_t(\mathbf {x}_2-\mathbf {z})$ around $\mathbf {x}_2$,

$$\begin{aligned} \psi ^I_t(\mathbf {x}_2-\mathbf {z})\approx \psi ^I_t(\mathbf {x}_2)-\nabla _{\mathbf {x}}\psi ^I_t(\mathbf {x}_2)\cdot \mathbf {z}+\frac{1}{2}\,\mathbf {z}^T\cdot \widehat{H}(\psi ^I_t(\mathbf {x}_2))\mathbf {z} \end{aligned}$$

where $\widehat{H}$ denotes the Hessian matrix. By replacing $\psi ^I_t(\mathbf {x}_2-\mathbf {z})$ in Eq. (10) by its Taylor expansion and considering that *W* is an even function of the components of the vector $\mathbf {z}=(z^1,z^2)$, we obtain

11 $$\begin{aligned} \psi ^I_{t+\epsilon }(\mathbf {x}_2)&=\psi ^I_t(\mathbf {x}_2)-\alpha (t)\psi ^I_t(\mathbf {x}_2)\,\epsilon +\nonumber \\&+\epsilon \psi ^S_t(\mathbf {x}_2)\int _{\Sigma }\,W(|\mathbf {z}|)\big [\psi ^I_t(\mathbf {x}_2))+\nabla _{\mathbf {x}}\psi ^I_t(\mathbf {x}_2)\cdot \mathbf {z}+\frac{1}{2}\,\mathbf {z}^T\cdot \widehat{H}(\psi ^I_t(\mathbf {x}_2))\mathbf {z}\big ]\,d^2\mathbf {z} \end{aligned}$$

12 $$\begin{aligned}&=\psi ^I_t(\mathbf {x}_2)-\epsilon \alpha (t)\psi ^I_t(\mathbf {x}_2)+\epsilon a \psi ^S_t(\mathbf {x}_2)\psi ^I_t(\mathbf {x}_2)+\epsilon D_t(\mathbf {x}_2)\,\nabla _{\mathbf {x}}^2\psi ^I_t(\mathbf {x}_2) \end{aligned}$$

where

$$\begin{aligned} a=\int _{\Sigma }\,W(|\mathbf {z}|)\,d^2\mathbf {z} \end{aligned}$$

and

13 $$\begin{aligned} D_t(\mathbf {x}_2)=\psi ^S_t(\mathbf {x}_2)\int _{\Sigma }\,W(|\mathbf {z}|)(z^1)^2\,d^2\mathbf {z}=\psi ^S_t(\mathbf {x}_2)\int _{\Sigma }\,W(|\mathbf {z}|)(z^2)^2\,d^2\mathbf {z}. \end{aligned}$$

Hence,

$$\begin{aligned} \frac{\partial \psi ^I_t(\mathbf {x}_2)}{\partial t}=(a\psi ^S_t(\mathbf {x}_2) -\alpha (t))\psi ^I_t(\mathbf {x}_2)+ D_t(\mathbf {x}_2)\nabla _{\mathbf {x}}^2\psi ^I_t(\mathbf {x}_2). \end{aligned}$$

Now, supposing the removed can be neglected with respect to the susceptible, we have $\psi ^S_t(\mathbf {x}_2)=\psi ^S_0(\mathbf {x_2})-\psi ^I_t(\mathbf {x_2})$ which, once replaced in the previous equation, gives

$$\begin{aligned} \frac{\partial \psi ^I_t(\mathbf {x}_2)}{\partial t}-D_t(\mathbf {x}_2)\nabla _{\mathbf {x}}^2\psi ^I_t(\mathbf {x}_2)=[(a\psi ^S_0(\mathbf {x}_2) -\alpha (t))-a\psi ^I_t(\mathbf {x}_2)]\psi ^I_t(\mathbf {x}_2). \end{aligned}$$

By renaming the terms we obtain

14 $$\begin{aligned} \frac{\partial \psi ^I_t(\mathbf {x}_2)}{\partial t}-D_t(\mathbf {x}_2)\nabla _{\mathbf {x}}^2\psi ^I_t(\mathbf {x}_2))=\eta \Big [1-\frac{\mu }{\eta }\psi ^I_t(\mathbf {x}_2)\Big ]\psi ^I_t(\mathbf {x}_2) \end{aligned}$$

where $\eta :=(a\psi ^S_0(\mathbf {x}_2)-\alpha )$ and $\mu =a$. Equation (14) is a two dimensional drift-diffusion equation which generalizes the Fisher–Kolmogorov equation and it is worth remarking that it has been derived from a non-linear probabilistic model.

A comment is in order since Eq. (14) describes the evolution of a probability density while the Fisher–Kolmogorov equation refers to the deterministic evolution of the population density. Nevertheless, assuming that the infected population is sufficiently large that the stochastic effects can be neglected, an analogous equation can be derived for the infected population density which, in this case, can be approximated by the expected number of infected $\rho ^I_t(\mathbf {x})=N\psi ^I_t(\mathbf {x})$. One obtains from (14),

15 $$\begin{aligned} \frac{\partial \rho ^I_t(\mathbf {x}_2)}{\partial t}-D_t(\mathbf {x}_2)\nabla _{\mathbf {x}}^2\rho ^I_t(\mathbf {x}_2))=\eta \Big [1-\gamma \rho ^I_t(\mathbf {x}_2)\Big ]\rho ^I_t(\mathbf {x}_2) \end{aligned}$$

where, $$\gamma =\frac{\mu }{N\eta }$$.

We obtain the Fisher–Kolmogorov equation by assuming $\alpha =0$ (which is equivalent to neglecting the removed) and by assuming $\psi ^S_t(\mathbf {x}_2)$ ($\rho ^S_t(\mathbf {x})$) constant and uniform. Those are reasonable assumptions if one is interested to study a single patch for sufficiently small time-intervals during which the susceptible are assumed to be uniformly distributed and the infected can be neglected with respect to the susceptible which can therefore be considered constant in time (the probabilistic model becomes linear under these hypothesis).

Thus, the Fisher–Kolmogorov equation is derived from a linear probabilistic model (the probability density of the susceptible is constant) and this extends previous results obtained by Mollison^34^ who showed that although the Fisher–Kolmogorov model is nonlinear, it is insufficient to grasp the full nonlinearity of the phenomena. In our opinion, it is relevant that a generalized version of the Fisher–Kolmogorov equation which is characterized by the time dependence of the diffusion coefficient (see Eqs. (14) and 15) is instead derived from a non-linear probabilistic model. That indeed suggests that the information about the non-linearity of the epidemic evolution could be fully encrypted in the time dependence of the diffusion coefficient $D_t(\mathbf {x})$ (anomalous diffusion); the latter being necessary for the probabilistic model to be non-linear (see Eq. (13)). It is worth remarking that anomalous diffusion has been recognized to be very relevant in many biological and physical systems. For example, it has been shown to be characteristic of the motion of single messenger RNA molecule in a living Escherichia coli bacterium^48^. A possible explanation for anomalous diffusion of the kind $D\propto t^\alpha$ in biological systems can be given by continuous-time random-walk models. The derivation of Eq. (14) from the non-linear probabilistic model seems to suggest another possible reason for anomalous diffusion, i.e., non-linearity of the probabilistic model. Moreover, Eqs. (13) seems to suggest that more general kinds of anomalous diffusion are possible. Those are problems deserving further investigation.

### Discretization of the model

Now we pass to consider the discretized version of the model. We obtain a general stochastic space-discrete model and, by neglecting the space effects, we obtain the SIR and SI models. If we discretize the time as well, we obtain a time-discrete stochastic model from which the stochastic SIR model can be derived.

#### Space discretization

Suppose we can divide $\Sigma$ into *n* subsets (not necessarily of the same size) $\{\Sigma _1,\dots ,\Sigma _n\}$. We will use the symbol $\Sigma =\{1,2,\dots ,n\}$ to denote this family of subsets and $\sigma _j$ to denote the area of $\Sigma _j$. A meaningful discretization procedure requires that the functions, $\psi ^I$, *W*, etc. are sufficiently regular and the subsets $\Sigma _j$ sufficiently small that $\psi ^I$, *W*, etc. can be considered constant in each subset $\Sigma _j$. The function $\psi ^I_t(\mathbf {x})$ is then replaced by the *n*-dimensional sub-stochastic vector $\widehat{p}^I(t)=(p_1^I(t),\dots ,p_n^I(t))$ where $p^I_j(t):=\sigma _j\psi ^I_t(j)$ is the probability at time *t* to find an infected in the region $\Sigma _j$. An analogous argument applies to the susceptible density which is replaced by the sub-stochastic vector $(p_1^S(t),\dots ,p_n^S(t))$. The discretized version of the kernel *W* is a $n\times n$ matrix $\widehat{W}_t=\{W_t(j,i)\}_{i,j=1,\dots ,n}$ and the evolution operator is obtained by multiplying Eq. (4) by $\sigma _j$,

16 $$\begin{aligned} p_j^I(t)&=p_j^I(0)-\int _0^t\,\alpha (t)p_j^I(\tau ) d\tau +\sum _{i=1}^n\int _{0}^{t}\,p_j^S(\tau )W_\tau (j,i)p_i^I(\tau )\,d\tau \\&=p_j^I(0)-\int _0^t\,\alpha (t)p_j^I(\tau )d\tau +\int _{0}^{t}p_j^S(\tau )(\widehat{W}_\tau \widehat{p}^I(\tau ))_j\,d\tau \nonumber \end{aligned}$$

where $\widehat{W}_\tau$ is the matrix $\{W_\tau (j,i)\}_{i,j=1,\dots ,n}$.

The evolution from time *t* to time $t+\epsilon$ is given by

17 $$\begin{aligned} p_j^I(t+\epsilon )&=p_j^I(t)-\int _t^{t+\epsilon } \alpha (t)p_j^I(t)\,dt+\int _t^{t+\epsilon } p_j^S(t)\sum _{i=1}^n \,W_t(j,i)p_i^I(t)\,dt\\ p_j^S(t+\epsilon )&=p_j^S(t)-\int _t^{t+\epsilon } p_j^S(t)\sum _{i=1}^n \,W_t(j,i)p_i^I(t)\,dt\nonumber \end{aligned}$$

where the second Eq. (17) is obtained by multiplying the discrete version of Eq. (7) by $\sigma _j$. By using again the Taylor expansion of $p_j^I(t+\epsilon )$ and $p_j^S(t+\epsilon )$ and assuming $\epsilon$ sufficiently small, we obtain

18 $$\begin{aligned} \frac{d p_j^I(t)}{d t}&=-\alpha (t)p_j^I(t)+p_j^S(t)\sum _{i=1}^n \,W_t(j,i)p_i^I(t) \nonumber \\&=-\alpha (t)p_j^I(t)+p_j^S(t)(\widehat{W}_t\widehat{p}^I(t))_j \end{aligned}$$

19 $$\begin{aligned} \frac{d p_j^S(t)}{d t}&=-p_j^S(t)\sum _{i=1}^n \,W_t(j,i)p_i^I(t) \nonumber \\&=-p_j^S(t)(\widehat{W}_t\widehat{p}^I(t))_j \end{aligned}$$

#### Space-time discretization and derivation of the SIR stochastic model

It is worth remarking that the discrete time version of Eqs. (17) provides a discrete time stochastic process. By choosing $\epsilon$ sufficiently small,

20 $$\begin{aligned} p^I_j(t+\epsilon )&=(1-\epsilon \alpha (t))p^I_j(t)+ p^S_j(t)\sum _{i=1}^n \,\epsilon W_t(j,i)p^I_i(t) \end{aligned}$$

21 $$\begin{aligned} p^S_j(t+\epsilon )&=p^S_{j}(t)-p^S_j(t)\sum _{i=1}^n \,\epsilon W_t(j,i)p^I_i(t) \end{aligned}$$

where $p^S_j(t)\sum _{i=1}^n \,\epsilon W_t(j,i)p^I_i(t)$ is interpreted as the probability that a new infected is produced during the time interval $\epsilon$. The process in now discrete in time with the time intervals that are multiples of $\epsilon$.

Once we have discretized the time, the SIR stochastic model^44–46^ (also known as the general stochastic epidemic) can be derived. Indeed, if for every region, we neglect the contribution from the other regions, $W_t(j,i)=\delta _{ij}\beta _i(t)$, we obtain

22 $$\begin{aligned} p^I_j(t+\epsilon )&=(1-\epsilon \alpha (t))p^I_j(t)+ p^S_j(t)\epsilon \beta _j(t)p^I_j(t) \end{aligned}$$

23 $$\begin{aligned} p^S_j(t+\epsilon )&=p^S_{j}(t)-p^S_j(t)\epsilon \beta _j(t)p^I_j(t) \end{aligned}$$

Now, let us denote by $n_j$ and $s_j$ the number of infected and susceptible in the region *j* respectively and assume $\epsilon$ sufficiently small to have $n_j(t+\epsilon )-n_j(t)\in \{-1,0,1\}$. Then, we can give the following interpretation of the probabilistic model: for every fixed *j*, $n_j$ and $s_j$ are random variables with expected values $Np^I_j$ and $Np^S_j$ respectively, the transition probability from the state $(n_j(t),s_j(t))$ to the state $(n_j(t)+1,s_j(t)-1)$ is given by $p^S_j(t)\,\epsilon \beta _j(t)p^I_j(t)$ while the transition probability from the state $(n_j(t),s_j(t))$ to the state $(n_j(t)-1,s_j(t))$ is given by $\epsilon \alpha p_j^I(t)$ and the transition probability from the state $(n_j(t),s_j(t))$ to the state $(n_j(t),s_j(t))$ is given by $1-p^S_j(t)\epsilon \beta _j p^I_j(t)-\epsilon \alpha p_j^I(t)$. Note that the transition probabilities are derived from the general model; they are not assumed by definition.

#### Derivation of SIR and SI models

Finally, we note that deterministic SIR and SI models can be obtained by neglecting the space effects. In order to show that, we assume *N* sufficiently large for the stochastic effects to be neglected and re-write Eqs. (18) in terms of the number of infected $n_j(t)=Np^I_j(t)$ and the number of susceptible $s_j(t)=Np^S_j(t)$ in the *j*-th region. We obtain,

24 $$\begin{aligned} \dot{n}_j(t)&=\frac{dn_j(t)}{dt}=N\frac{d p_j^I}{d t} \end{aligned}$$

25 $$\begin{aligned}&=-N\alpha (t)p^I_t(j)+Np^S_j(t)\sum _{i=1}^n \,\frac{1}{N}W_t(j,i)Np^I_t(i)\big ] \end{aligned}$$

26 $$\begin{aligned}&=-\alpha n_j+s_j\sum _{i=1}^n\Pi _t(j,i)n_i \end{aligned}$$

27 $$\begin{aligned} \dot{s}_j(t)&=\frac{ds_j(t)}{dt}=N\frac{d p_j^S}{d t} \end{aligned}$$

28 $$\begin{aligned}&=-s_j\sum _{i=1}^n\Pi _t(j,i)n_i \end{aligned}$$

where, $$\Pi _t(j,i)=\frac{1}{N}W_t(j,i)$$.

If we neglect the contribution of the other regions, i.e., $\Pi _t(j,i)\approx \delta _{ji}c_{j}(t)$, (which means that we are neglecting the space effects on the evolution of the epidemic), the system of differential equations becomes

29 $$\begin{aligned} \dot{n}_j(t)&=-\alpha n_j(t)+s_j(t)c_{j}(t)n_j(t) \end{aligned}$$

30 $$\begin{aligned} \dot{s}_j(t)&=-s_j(t)c_{j}(t)n_j(t) \end{aligned}$$

which coincides with the equations for infected and susceptible of an SIR model with time-dependent parameters $c_j(t)$, $\alpha (t)$ and with birth rate and death rate equal to zero (we assume that deaths and births compensate each other). SIR models are particular cases of the stochastic model we have introduced. If moreover, we neglect the removed, we obtain an SI model

31 $$\begin{aligned} \dot{n}_j(t)&=s_j(t)c_{j}(t)n_j(t) \end{aligned}$$

32 $$\begin{aligned} \dot{s}_j(t)&=-s_j(t)c_{j}(t)n_j(t) \end{aligned}$$

In particular, the relation between infected and susceptible is $s_j(t)=s_j(0)-n_j(t)$ where $s_j(0)$ is the number of susceptible at time $t=0$ and the equation for the infected becomes

$$\begin{aligned} \dot{n}_j(t)&=s_j(0)c_{j}(t)n_j(t)-c_{j}(t)n^2_j(t)\\&=kn_j(t)-\frac{k}{a}n^2_j(t) \end{aligned}$$

where $k=c_j(t)s_j(0)$ and $a=s_j(0)$, which is a logistic equation with time-dependent parameters. Assuming $c_j(t)$ time-independent we obtain the Verhulst logistic Eq. ^47^ (Verhulst 1838) whose solution is the sigmoid function

$$\begin{aligned} n_j(t)=\frac{a}{1+q e^{-kt}} \end{aligned}$$

where $q=\frac{a-n_j(0)}{n_j(0)}$ and $n_j(0)$ is the number of infected at time $t=0$.

## Discussion

We have proposed a general space-time continuous probabilistic model where the state of the epidemic is given by three sub-probability density functions. The latter are interpreted by analogy with the statistical interpretation of quantum mechanics. Then, we showed that many important stochastic and deterministic models can be derived as particular cases. That should be helpful from the theoretical viewpoint since it can be used to analyze further the relationships between different modelling approaches as well as their limits. For example, it has been shown that the non-linearity of the Fisher–Kolmogorov equation is not sufficient to characterize the non-linearity of the stochastic phenomena it aims to approximate^33, 34^. We strengthen and generalize such a result but also show that a general version of the equation characterized by anomalous diffusion (the diffusion coefficient is space-time dependent) is more deeply connected to the non-linearity of the probabilistic model it is derived from. That is of general interest from the physical viewpoint as well as for biological applications since it concerns the relationships between non-linear stochastic and non-linear deterministic models. Moreover, anomalous diffusion plays a relevant role in the diffusion of single messenger RNA molecules in living cells and in many other physical and biological systems^48–51^. A possible explanation for anomalous diffusion of the kind $D\propto t^\alpha$ in biological systems can be given by continuous-time random-walk models. The derivation of Eq. (14) from the non-linear probabilistic model seems to suggest another possible reason for anomalous diffusion, i.e., non-linearity of the probabilistic model; it furthermore suggests that more general kinds of anomalous diffusion could be possible. A future work will be devoted to push forward such investigations.

Possible applications to Covid-19 epidemic concern the time evolution of the probability density $\psi ^I_t$. The density $\psi ^I_t$ can be approximated by the kernel density estimation method (KDE) and its evolution is determined by Eqs. (6) and (8). The knowledge of such evolution, which is encoded into the kernel *W*, would allow probabilistic epidemic forecasting; it would allow to locate those regions of space where the probability of the infection is going to increase. All of that depends on our capacity to estimate the transition kernel *W*. It is a kind of inverse problem that requires insight into the real scenarios. Concerning this last point, some preliminary work^52^ that could be helpful and some applications^53^ of the kernel density estimation method based on ideas that could be relevant, already exist but much more effort is necessary in order to estimate *W* starting from data. More mathematical work is required as well. That will be the topic of a future work.
